# Graph-Based Motion Artifacts Detection Method from Head Computed Tomography Images

**DOI:** 10.3390/s22155666

**Published:** 2022-07-28

**Authors:** Yiwen Liu, Tao Wen, Wei Sun, Zhenyu Liu, Xiaoying Song, Xuan He, Shuo Zhang, Zhenning Wu

**Affiliations:** 1 School of Computer Science and Engineering, Northeastern University, Shenyang 110819, China; wentao@neusoft.edu.cn; 2 Department of Computer Science and Technology, Dalian Neusoft University of Information, Dalian 116023, China; liuzhenyu@neusoft.edu.cn (Z.L.); songxiaoying@neusoft.edu.cn (X.S.); 3 School of Computer Science, Neusoft Institute Guangdong, Foshan 528225, China; sunwei@neusoft.edu.cn; 4 College of Medicine and Biological Information Engineering, Northeastern University, Shenyang 110819, China; hexuan@bmie.neu.edu.cn; 5 School of Information Science and Engineering, Northeastern University, Shenyang 110819, China; 1910307@stu.neu.edu.cn (S.Z.); wuzn2003@hotmail.com (Z.W.)

**Keywords:** complex networks, computed tomography images, motion artifacts detection, network topological characteristics, classification

## Abstract

Computed tomography (CT) images play an important role due to effectiveness and accessibility, however, motion artifacts may obscure or simulate pathology and dramatically degrade the diagnosis accuracy. In recent years, convolutional neural networks (CNNs) have achieved state-of-the-art performance in medical imaging due to the powerful learning ability with the help of the advanced hardware technology. Unfortunately, CNNs have significant overhead on memory usage and computational resources and are labeled ‘black-box’ by scholars for their complex underlying structures. To this end, an interpretable graph-based method has been proposed for motion artifacts detection from head CT images in this paper. From a topological perspective, the artifacts detection problem has been reformulated as a complex network classification problem based on the network topological characteristics of the corresponding complex networks. A motion artifacts detection method based on complex networks (MADM-CN) has been proposed. Firstly, the graph of each CT image is constructed based on the theory of complex networks. Secondly, slice-to-slice relationship has been explored by multiple graph construction. In addition, network topological characteristics are investigated locally and globally, consistent topological characteristics including average degree, average clustering coefficient have been utilized for classification. The experimental results have demonstrated that the proposed MADM-CN has achieved better performance over conventional machine learning and deep learning methods on a real CT dataset, reaching up to 98% of the accuracy and 97% of the sensitivity.

## 1. Introduction

Computed tomography (CT) is widely used for diagnosis of various disease for the sake of its readily accessibility and low cost. However, artifact is one of the commonly encountered problems in CT, which can be generally described as any non-consistency between the real image and the reconstructed image, resulting from devices and individuals. It may significantly degrade the image quality, cause harm to clinical diagnosis and compromise the medical image post-processing. Therefore, it is essential to detect artifacts from CT images [1,2]. To automatically detect artifacts, many efforts have been made over the past decades. To comprehensively understand artifacts formation mechanism, researchers did a plenty of valuable work. Spin-Neto et al. [3] has placed a strong emphasis on explaining the physics behind the occurrence of motion artifacts with the aim of aiding artifact detection and mitigation in clinical situations. The impact and the correction of motion artifacts on diagnostic accuracy of apical periodontitis in CBCT images have also been demonstrated in [3]. To build dental implant artifact detection algorithm, seven designated features were extracted from ROIs (regions of interest) and machine learning with random forests was used in [4]. Recently, deep-learning methods have experienced a significant growth, they can extract useful information from underlying data [5,6] in the field of computer vision [7] and have shown unprecedented competitiveness in image classification [8], a convolutional neural network (CNN)-based tool has been used for identifying motion artifacts in various magnetic resonance imaging [9,10]; moreover, dental artifacts on each slice of a HN CT scan have also been detected effectively [11].

Although CNNs have gained a great reputation and received substantial attention both in computer vision community and other fields such as medical imaging applications [11,12,13], there are still some limitations in the clinical practice. CNNs merely take physical features into account and pay little attention to the relationship between the network topological properties of different images. Moreover, due to the difficulty of collecting CT images with artifacts in clinical practice, there are generally few datasets with sufficient samples for model training in the clinical practice.

Complex Network theory [14,15,16] is a powerful method for describing the complexity of systems [17]. Due to its flexibility and generality, the representation of different structures as a graph by modeling a set of nodes linked with edges has been widely conducted [18]. Recently, researchers have described and explained the complex interactions among components in the complex systems, such as economics [19], biology [20], social sciences [21], and the earthquake network [22,23], from a novel perspective. By analyzing graphs, the insights of the structure and the corresponding network topological characteristics, such as average shortest path, clustering coefficient, degree distribution, have been studied widely in various complex networks [24,25]. In addition, the complex network is a low complexity and effective method to deal with large-scale data and their relations based on graph theory. Motivated by complex network theory, images have begun to engage with the literature of complex networks. Methodologies based on complex networks to better characterize an image considering topological feature have been carried out as a notable paradigm. However, few researchers have already introduced the complex network theory into the detection of motion artifacts and topological features have not been taken into consideration for detecting motion artifacts from CT images.

For medical image quality assess and artifacts detection [26,27,28], there are still three major challenges remaining.

The first challenge is the effectiveness of feature extraction. Current approaches to CT motion artifacts detection may not obtain the characteristics of the image thoroughly as topological properties have not been involved.

Since accessible medical databases are limited, and valid medical ground truth databases for the evaluation of algorithms are rare and usually comprise only a few images [29]. The effectiveness of CT motion artifacts detection with limited data is still far from reach.

Another challenge with regards to the current motion artifacts detection method is how to explain the acquired results that meet the demand of radiologists but fail to meet clinical requirements. At present, in-depth studies on the mechanisms causing artifacts are lacking.

Based on the above discussions, in this paper, a new framework based on complex networks has been proposed to explore the hidden information behind the CT images, extract topological features for interpretability and detect motion artifacts. The main contributions of this work are summarized as follows:

Motion artifacts detection problem has been reformulated as a complex network classification problem based on network topological characteristics.

An interpretable graph-based motion artifacts detection method has been proposed in clinical practice. Two-level classification has been proposed to detect motion artifacts effectively.

Extensive experiments have been carried out on a real-world dataset and experimental results achieve comparable performance on evaluation criteria, in terms of motion artifacts detection, which meet the medical clinical demand to a larger extent.

## 2. Materials and Methods

### 2.1. Data and Basic Assumptions

#### 2.1.1. Dataset

Artifact is applied to any systematic discrepancy between CT numbers in the reconstructed image and the true attenuation coefficients of the object, it can seriously degrade the quality of CT images; in particular, motion artifacts are caused by patient movement, which mostly appear as shading or streaking in the reconstructed image [30]. A single CT scan has several slices.

**Figure 1:**
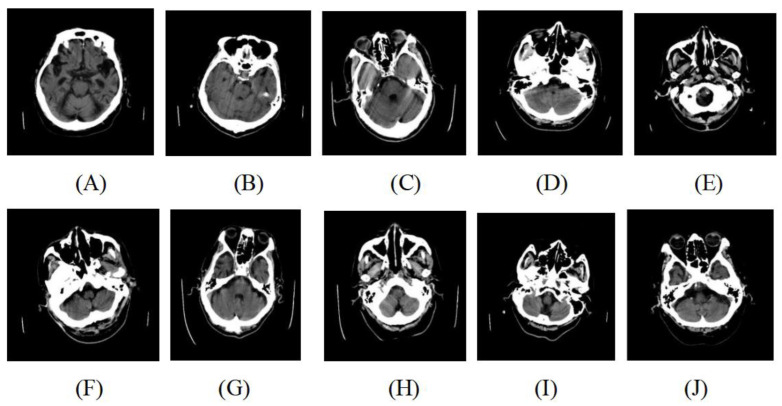
Illustration of original slices. (**A**–**E**) represent slices with motion artifacts. (**F**–**J**) represent slices without motion artifacts.

The dataset containing 600 head CT slices collecting from Neusoft Medical CT scanner is used in this paper. The ground-truth of head CT slices are labeled as a binary classification problem, including 300 positive samples (with motion artifacts) and 300 negative samples (without artifacts) by a neuroradiologist with 7 years’ experience. The resolution of each slice is 128 × 128. Examples of head CT slices are illustrated in Figure 1.

#### 2.1.2. Assumptions

Motivated by medical image analysis, for example MRI, based on complex network theory, which has advantages in handling unstructured data, graph construction based on complex network theory may also achieve better performance when utilized for CT images quality analysis.

### 2.2. Complex Network-Based Graph Construction

A mathematical graph representation of the head CT image network is given by a simple graph as follows:

(1) G=(V,E)

*V* = $\left\{v_{1},v_{2}\;,\;\ldots \;,\;v_{n}\right\}$ is the set of vertices, *E =* {*e_ij_*|*i* ∈ *V*, *j* ∈ *V*, *e_ij_* ∉ 0} is the set of edges.

For *e_ij_ = e_ji_*, the head CT images network is undirected.

As a weighted network is often used in the study of the interactive process, an unweighted network is suitable to explore network structure. Since the scale of the dataset is fixed, we use unweighted graph to represent head CT image.

In summary, the head CT image can be mapped as an undirected, unweighted network.

For the sake of the comprehensiveness and specificity, we design a two-level graph construction method based on the complex network theory; our process of constructing pixel-level and slice-level graph is as follows.

#### 2.2.1. Pixel-Level Graph Construction Based on the Complex Network Theory

At pixel level, the edges represent the mutual relationships between vertices, the edges are generated according to the location and grey scale between two pixels.

To make the connections between the vertices, pixel-by-pixel sweep over the image is performed, grey difference has been calculated between its neighbors’ pixels for each pixel selected. If distance between two pixels is not bigger than a given radius r (here, we set r = 3), the two vertices are connected by an edge.

#### 2.2.2. Slice-Level Graph Construction Based on the Complex Network Theory

At slice level, all of the slices have mapped network topology based on the complex network theory to represent the mutual relationships between slices, and the edges are generated when two slices of the same region sharing the same label. In this way, the complex slice-slice correlation has been replaced by the edge. Slice-level graph construction considers the heterogeneity of individuals, it has been pointed out that the correlation-based methods provide relatively higher sensitivity of the network connection [31].

**Figure 2:**
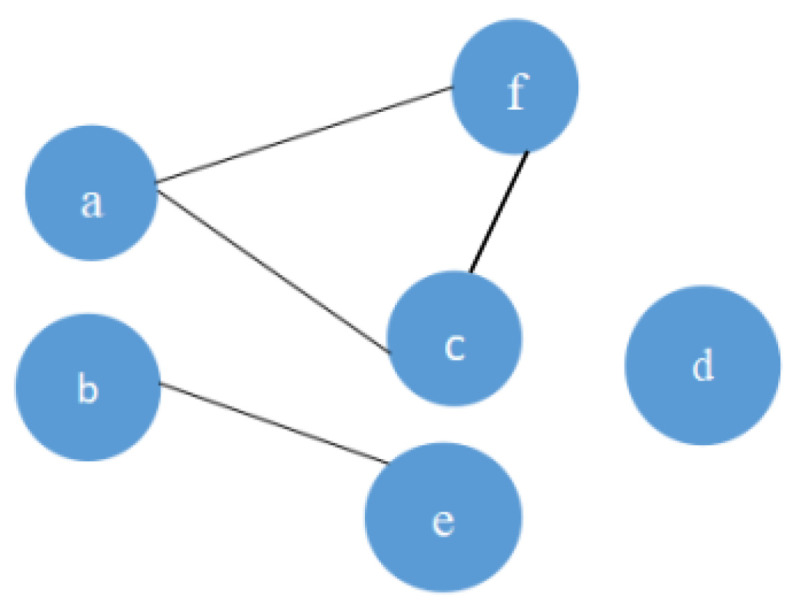
A schematic description of slice-level networks based on region and label consistency. Edges which are described by the straight lines are generated when the slices belonging to the same region label share the same quality label. Otherwise, the two slices are considered to be independent to each other.

In order to describe the above method, we first obtain a set of data from a real dataset as shown in Table 1. As the head CT images contains different slices reconstructed from different angles, the regions are essential and of great important for the recognition and exploration for classification. Actually, region is an attribute of slice. The slices which share the same region may have similar features in nature than those with different regions; that is to say, the slices are of great difference in nature according to the region they belong to. We apply the data to generate related network topology, as shown in Figure 2. CT slices are denoted as {a, b, c, d,e,f}, the edge is represented as straight lines.

By the two-level graph construction based on the complex network theory, both local information and the complementary information among multiple CT slices have been taken into account.

### 2.3. Basic Network Topological Properties

The network topological properties [21] are explained in detail.

Vertex Degree [14]: the degree is the most fundamental character and measure of a vertex. The degree of a given vertex *i* is the number of vertices connected to it for an undirected graph.

Average Degree: the average degree of *G* is the sum from the graph’s number of edges divided by its number of vertices.

Degree Distribution: the degree distribution is of great important in *G*. The degree distribution is defined by a probability function, *P*(*k*), which can be understood as the probability that a randomly picked vertex has degree *k*.

Clustering coefficient [16]: clustering coefficient is the coefficient used to describe the degree of clustering between the vertices of a graph. It implies the degree of clustering of the network. In a graph, let *i* be a vertex with *k_i_* edges, which are called neighbors of vertex *i*. Let *deg*(*i*) be the number of the actual edges existing among the *k_i_* vertice. The ratio between the actual and the possible numbers of edges in the cluster of the *k_i_* vertice is defined to be the clustering coefficient of the vertex *i*, denoted as *C_i_*

(2) $$C_{i}=\frac{2deg\left(i\right)}{k_{i}\left(k_{i}-1\right)}$$

The network topological properties may indicate the underlying organization principles of head CT images and help in understanding possible relevance of CT image quality. The notations of basic network topological properties have been illustrated in Table 2.

Single topological property can only describe the feature of head CT images from one particular aspect. It is necessary to combine different topological properties to comprehensively understand the head CT images. In this way, we conduct multi-dimension topological properties and consistency investigation.

### 2.4. Motion Artifacts Detection Method Based on Complex Networks (MADM-CN)

**Figure 3:**
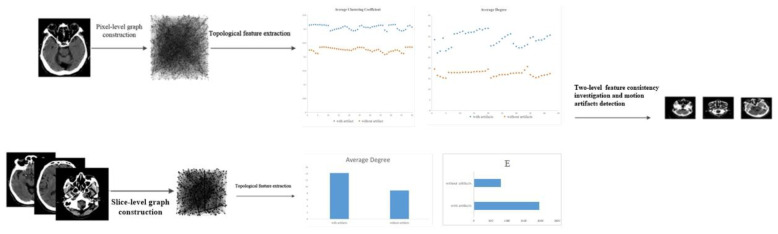
Pipeline of Motion Artifacts Detection Method based on topological properties.

The framework of the proposed motion artifacts detection method based on complex networks (MADM-CN) is illustrated in Figure 3. The general pipeline of the method is described as follows:

#### 2.4.1. Feature Extraction and Selection Based on the Complex Networks

On the one hand, based on the pixel-level graph construction, the head CT images with and without artifact have been comprehensively expressed since pixel-to-pixel relationships are represented as graphs. From the perspective of the complex network, the network topological properties are exploited to express meta head CT image locally.

On the other hand, based on the slice-level graph construction, the head CT images with and without artifact have been comprehensively expressed since image-image relationships are represented as graphs. From the perspective of complex network, the network topological properties are measured from head CT slices globally.

Based on the two-level graph construction method, network topological characteristics including the number of edges, average degree, average clustering coefficient can be investigated, pixel-level and slice-level topological characteristics analysis are investigated locally and globally; for pixel level analysis we focus on pixel-to-pixel relationship in a single CT image while for slice-level analysis we focus on slice-to-slice relationship in the datasets. Furthermore, the vertex of slice-level graph is the single CT image. Two-level feature extraction method are utilized to extract consistency two-level robust features. In this way, a combination of node attribution and graph properties has been made for motion artifacts detection.

#### 2.4.2. Classification

For a judgement problem with two classes, images with artifact and without artifact are established as positive and negative, respectively. When it comes to the motion artifact detection step, classifiers, machine learning methods, such as Support Vector Machine (SVM) and Random Forest (RF), and deep learning methods, such as CNN, have been used for classification. In particular, we use SVM(RBF kernel), RF(1000 trees) and ResNet for classification.

#### 2.4.3. Motion Artifacts Detection and Evaluation

To validate the effectiveness of the proposed MADM-CN method, a real-world dataset would be utilized to detect motion artifacts from head CT images. From a clinical practice perspective, sensitivity, which is an indicator that radiologists care the most, would be used for evaluation. Moreover, accuracy and specificity have also been taken into consideration.

## 3. Results

### 3.1. Graph Construction

**Figure 4:**
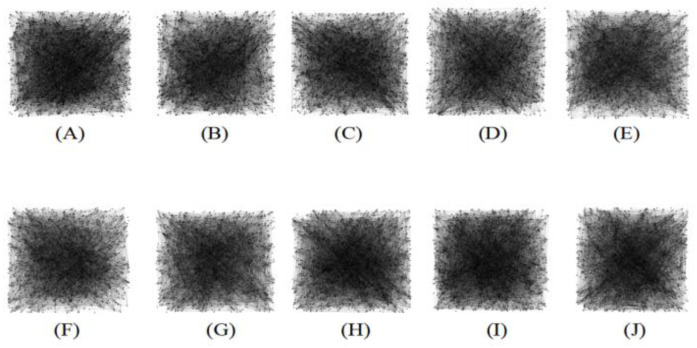
Corresponding graphs generated based on pixel-level graph construction. (**A**–**E**) represent head CT images with motion artifacts. (**F**–**J**) represent head CT images without artifacts.

At pixel level, after graph modeling, we obtained corresponding graphs (See Figure 4A–J).

For the constructed graph, we obtain topological properties including average degree, average clustering coefficient from CT images with and without artifacts, respectively.

**Figure 5:**
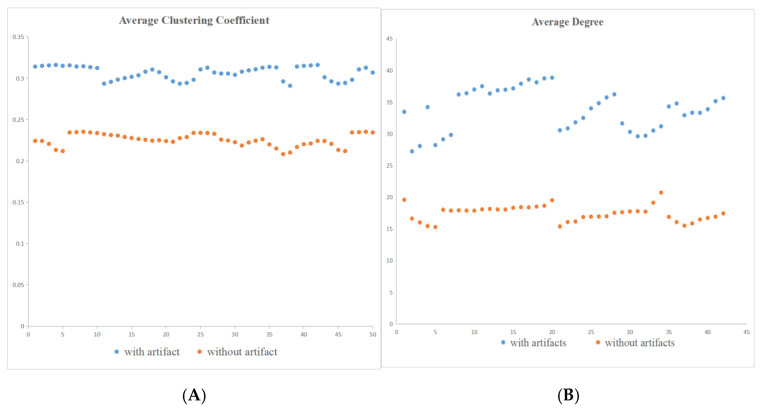
Different network topological properties from a part of data sets, (**A**) depicts the average clustering coefficient, (**B**) depicts average degree, respectively. It is worth noting that both the average clustering coefficient and the average degree of the CT images with artifacts are larger than those of the CT images without artifacts, respectively.

As shown in Figure 5, the multi-dimension network topological properties including average clustering coefficient and average degree have stable performance, which means that they have the potential to be applied for subsequent classification. From the topological perspective, at pixel level, motion artifacts form due to the increase in average clustering coefficient and average degree.

**Figure 6:**
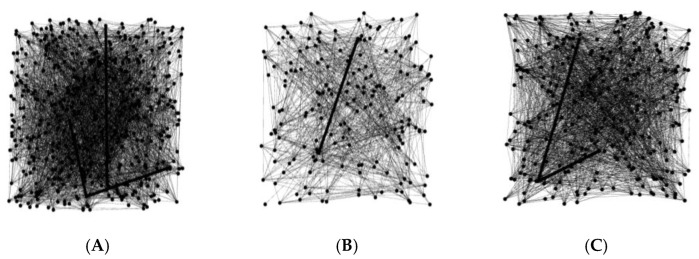
The constructed graphs using slice-level graph construction method. (**A**) is the hybrid graph which consists of head CT slices with motion artifacts and head CT slices without artifacts, (**B**) contains head CT slices without artifacts alone, (**C**) contains head CT slices with artifacts alone.

At slice level, after graph construction, we have obtained the corresponding graphs in Figure 6.

The constructed graphs representing different head CT slice qualities help to understand the artifacts formation mechanism in a topological way. The topological properties are explored in Table 3.

Combined with Figure 5, the topological properties have possible relevance of different CT slice qualities, taking the network topological properties stability and local-global consistency into consideration, average clustering coefficient and average degree have been selected for motion artifacts detection.

### 3.2. Performance Metrics

For a judgement problem with two classes, CT slices with artifact and without artifact are established as positive and negative, respectively. Metrics of artifactes detection judgement have been illustrated in Table 4. From the perspective of clinical application, since radiologists are more concerned about whether all artifact images are detected, sensitivity is regarded to be the most important metric. In artifacts detection, the higher sensitivity indicates the higher probability that artifact image is detected.

(3) $$Accuracy=\frac{TP+TN}{TP+FP+FN+TN}$$

(4) $$Specificity=\frac{TN}{FP+TN}$$

(5) $$\;Sensitivity=\frac{TP}{TP+FN}$$

### 3.3. Experimental Results

We split the dataset into training set and test set at a ratio of 80%:20%.

We evaluate the model performance by accuracy, sensitivity, and specificity and AUC on test set. We compare detection performance as shown in Table 5; the proposed method works well in the clinical practice.

To evaluate the impact of topological properties utilization for classification, the comparison has been conducted. We evaluated two classifiers (SVM, RF) to predict whether CT slices are artifacts-affected or not when taking topological characteristics into account. In addition, we also compared with the DL-based baseline approach, CNN.

From Table 5, we can see that by using physical and topological properties in combination we achieve the highest accuracy, sensitivity and AUC with the proposed MADM-CN model employing SVM. We also conclude that including the topological properties into the training (as well as the physical characteristics) improves the performance.

It can also be seen from Table 5 that the results of traditional algorithms, SVM and RF with physical and topological properties outperform the state-of-the-art motion artifacts detection method, CNN, and those with merely physical features with limited sample data, in particular, our proposed MADM-CN method achieves highest accuracy using SVM with limited samples. When it comes the sensitivity, our method proposed MADM-CN performs best when employing SVM. The MADM-CN method obtains the accuracy of 98% and the sensitivity of 97% when it employs SVM for classification.

It has also been indicated that topological properties are robust and can embed comprehensive description to fully express the characteristics of the head CT slices with and without artifacts, respectively. In addition, average degree and average clustering coefficient have been applied resulting in higher accuracy and sensitivity. The topological properties indicate the internal mechanism of head CT image formation. It can be demonstrated that the head CT slices with motion artifacts have larger average clustering coefficient and average degree than those without motion artifacts. The head CT slices with small average clustering coefficient and average degree may have better quality.

## 4. Discussion and Conclusions

In this paper, an interpretable graph-based motion artifacts detection method has been proposed. We reformulate the artifacts detection problem as a complex network classification problem based on network topological properties. A comprehensive description of head CT images has been worked out to effectively express the characteristics of head CT images with and without artifact, respectively. First, CT images have been constructed into graphs at pixel and slice level, respectively. Then, based on the topological measurements, it is found that average clustering coefficient and average degree are closely correlated to the image quality locally and globally. Finally, when applying the proposed methods on a real dataset of head CT images, motion artifacts has been effectively detected.

Our results confirm that the MADM-CN method can identify the quality of the head CT images more effectively in clinical practice. It shows high accuracy and sensitivity even with limited labeled samples, which meets clinical demand to a larger extent.

An interpretable graph-based framework has been designed to detect motion artifacts from head CT images. A two-level graph construction method based on complex network theory is proposed. The topological properties indicate the underlying organization principles of different CT image qualities.

It can be demonstrated that the head CT images with motion artifacts may have larger average clustering coefficient and average degree than those without motion artifacts. Head CT images with a small average clustering coefficient and an average degree may have better quality.

The study of motion artifacts detection from the perspective of the complex network will help radiologists to study the law of artifacts formation mechanism in a new way.

Currently, the graph-based method exclusively detects motion artifacts from head computed tomography; as the method explores the characteristics of medical images, it can be used for other head CT artifacts, for instance, metal artifacts [32] in the maxillomandibular complex [29] and may be suitable for other modalities. Since the region label has been marked manually, an automatic region detection method will be taken into account for a future direction.

## Figures and Tables

**Table 1:** Head CT Slices of different qualities in different regions.

Slice ID	Region Label	Quality Label
a	1	0
b	2	1
c	1	0
d	3	1
e	2	1
f	1	0

**Table 2:** The Notations of basic network topological properties.

Notation	Implication
V	The set of vertices
E	The set of edges
Average Degree	The sum from the graph’s number of edges divided by its number of vertices.
Average Clustering Coefficient	The degree of clustering of constructed network

**Table 3:** The network topological properties of slice-level graph.

Dataset	N	Average Clustering Coefficient	Average Path Length	Average Degree	|E|
Hybrid CT images	600	0.994	1.127	12.039	2082
CT images without artifacts alone	300	0.988	1.357	8.817	821
CT images with artifacts alone	300	0.998	1.006	14.186	1981

**Table 4:** Metrics of artifacts detection judgement.

		Predicted	
		1	0
True	1	True Positive (*TP*)	False Negative (*FN*)
False	0	False Positive (*FP*)	True Negative (*TN*)

**Table 5:** Performance compared with the state-of-the-art methods.

Classification	Features	Level	Sensitivity	Accuracy	Specificity	AUC
MADM-CN + SVM	Physical + Topological	hybrid	97%	98%	96%	0.9668
MADM-CN + RF	Physical + Topological	hybrid	95%	97%	98%	0.9591
CNN	Physical	Pixel	86.67%	76.67%	66.67%	0.722
RF	Physical	Pixel	85%	88%	89%	0.9366
SVM	Physical	Pixel	80%	88%	93%	0.8819

## Data Availability

The dataset used and/or analyzed during the current study is available from the corresponding author upon reasonable request. The dataset is not publicly available due to privacy.
